# Dietary behaviors and attitudes among Norwegian medical students

**DOI:** 10.1186/s12909-023-04194-4

**Published:** 2023-04-06

**Authors:** Ingebjørg Sanne, Anne-Lise Bjørke-Monsen

**Affiliations:** 1 Department of Hematology and Oncology, Stavanger University Hospital, Stavanger, Norway; 2 Laboratory of Medical Biochemistry, Innlandet Hospital Trust, Lillehammer, Norway; 3 Laboratory of Medical Biochemistry, Førde Central Hospital, Førde, Norway; 4 Department of Medical Biochemistry and Pharmacology, Haukeland University Hospital, Bergen, 5021 Norway

**Keywords:** Nutrition education, Medical students, Food attitudes, Food marketing

## Abstract

**Background:**

Dietary patterns and beliefs are influenced by socioeconomic status, cultural influences, as well as medical advice, social media and marketing. Medical doctors are expected to provide correct, updated and non-biased nutritional advice to their patients, but their own dietary behaviors and attitudes may influence nutritional counselling.

**Methods:**

We have investigated dietary habits and food attitudes among medical students by using an anonymous survey distributed to all students at the Medical Faculty, University of Bergen, Norway. The survey included a 36-item questionnaire covering information about demographics, former and current diet, use of nutritional supplements, tobacco and alcohol, in addition to food attitudes and nutritional knowledge. Descriptive statistics were calculated for each survey item.

**Results:**

Of the 880 students, 394 responded to the survey. Although 90% of the students were omnivores, the majority had a negative attitude towards meat, and considered fish to be healthier than meat. Significantly more women than men reported use of a special diet and excluded meat from their diet, even if they were omnivores. The most frequently used supplement was cod liver oil or omega 3 fatty acids.

**Conclusion:**

The medical students’ diet and food attitudes not only reflect current health recommendations, but also popular beliefs and marketing in Norway. Curriculum planners should make the students capable of recognizing the influence of social media, marketing and medicine-food industry interactions, to ensure relevant nutrition knowledge for future doctors.

## Background

A balanced diet is important for maintaining optimal health, growth and development, and medical doctors are expected to provide correct and updated nutritional advice to their patients. For decades a mixed, balanced diet including both plant and animal foods was the dominant nutritional regime, but this is now being challenged [1, 2]. New dietary patterns are quickly evolving [3], so it may be more demanding than before for doctors to give correct nutritional counselling.

Nutrition receives a substantial amount of media coverage, and food promotions are found to have a direct effect on nutrition knowledge, preferences, purchase behaviour, consumption patterns and diet-related health [4]. Nutraceuticals, defined as food or food substances, which provide medical or health benefits [5], have a global market and are being promoted through various channels, including research and clinical trials [6]. Food industry-sponsored nutrition research is prone to produce results that confirm health positive effects of the product [7] and medical doctors need to be aware of the concept of addressing conflicts of interest in nutritional advices. According to a recent review, healthcare professionals should routinely screen their patients for current eating habits and educate them about diet and health risks [8].

As healthy diet-related habits and food attitudes have been related to physicians’ likelihood to counsel their patients about nutrition and weight [9, 10], we wanted to study dietary habits and food attitudes among future doctors and conducted a survey among Norwegian medical students. Also, food attitudes in a highly health-educated population, such as medical students, may provide a benchmark for health habits and food attitudes in young adults [11]. Current Norwegian dietary guidelines recommend a varied diet with an increased amount of vegetables, fruit and fish and a reduced amount of red meat [12]. Do medical students follow these recommendations and are their diet and food attitudes influenced by popular beliefs and media marketing?

## Methods

### Study population and design

In the fall semester of 2018, medical students (n = 880) at the University of Bergen were invited to take a survey on nutritional knowledge. The survey included a 36-item questionnaire covering information about demographics, former and current diet, use of nutritional supplements, tobacco and alcohol, in addition to food attitudes and nutritional knowledge. One article covering nutritional knowledge among the medical students based on this survey has been published[13]. The survey was created using the online survey tool SurveyMonkey (SurveyMonkey.com, LLC, Palo Alto, California, USA) and advertised at the website of the Medical Faculty, University of Bergen. All data was collected anonymously, and all methods were performed in accordance with the relevant guidelines and regulations. The study was approved by the Norwegian Centre for Research Data (NSD), reference 108,552.

### Assessment of dietary practice, food attitudes and nutritional knowledge

The students were asked about ethnicity, age, weight, height, physical exercise, as well as use of alcohol, tobacco and nutritional supplements[13]. They were asked if they adhered to one of the following diets: omnivore, including Fodmap, gluten-free, low-carb, stone age, vegetarian, (i.e. lacto-vegetarian: plant food in addition to dairy products and lacto-ovo-vegetarian: also including egg), or vegan (plant food only). A Fodmap diet has a reduced amount of fermentable oligosaccharides, disaccharides, monosaccharides and polyols, carbohydrates which are difficult to digest.

The students were asked about their dinner choices and if they avoided specific foods, and if so, the reason for this. Food attitudes concerning meat, fish and vegetarian diets were tested by agreement to different statements.

Clarity of the questions, accuracy of the knowledge measured, and interpretability were assessed by an expert of nutrition. The survey was applied only once to each student, so we did not have the possibility to assess whether the results were replicable.

### Statistical analysis

Descriptive statistics were calculated for each survey item. Continuous data are presented as mean and standard deviation (SD) compared Student’s t-test. Categorical data are presented as numbers (%) and compared by Chi-square test.

Two-sided p-values < 0.05 were considered statistically significant. The SPSS statistical program (version 25) was used for the statistical analyses.

## Results

### Demographics

A total of 394 of the 880 (45%) students responded to the survey. Baseline demographic characteristics of the included students are shown in Table 1. The students were evenly distributed among the six years of the medical school and the majority were of European origin (94%). The students were mainly young people (range 18–47 years), only 26% were married or cohabitants and 5% had children.

Most students (82%) had a normal BMI (18.5–25), 3% were underweight (BMI < 18.5) and 15% were overweight (BMI > 25). All but one of the underweight students were female and significantly more male students (29%) were overweight compared to females (12%), p < 0.001. The students reported regular exercise (91%), with an average of 4.2 h of exercise per week (Table 1). Regular use of tobacco was uncommon (1%), whereas regular use of alcohol was common (82%).

**Table 1:** Baseline characteristics of the medical students (n = 394)

Parameters	Women N = 313	Men N = 81	P value
Age, y, mean (SD)	23.6 (4.3)	23.6 (3.4)	0.39^a^
BMI, kg/m^2^, mean (SD)	22.1 (3.1)	23.3 (3.5)	< 0.001^a^
Regular exercise, n (%)	285 (91%)	74 (91%)	0.93^c^
Hours per week, mean (SD)	4.0 (3.0)	4.0 (4.0)	0.07^a^
Regular use of tobacco, n (%)			
Cigarettes	2 (1%)	2 (3%)	0.14^b^
Snuff	31 (10%)	11 (14%)	0.56^b^
Regular use of alcohol, n (%)	256 (82%)	68 (84%)	0.65^b^
Units per week, mean (SD)	2.0 (3.5)	2.5 (3.8)	0.10^a^
Current diet, n (%)			
Omnivore	275 (88%)	79 (98%)	0.01^b^
Vegetarian	30 (12%)	2 (3%)	0.01^b^
Vegan	8 (3%)	0 (0%)	0.15^b^
Regular use of supplements, (≥ 3 days/week), n (%)			
Multivitamins/minerals	99 (31%)	10 (12%)	0.001^b^
Iron	48 (15%)	2 (3%)	0.002^b^
Fish oil	70 (22%)	22 (27%)	0.36^b^
Omega 3 fatty acids	79 (25%)	16 (20%)	0.30^b^

^a^ Comparison by Student’s t-test

^b^ Comparison by Pearson Chi-Square test

### Diet

Most of the students (n = 354, 90%) reported having a current omnivore diet, while 40 students (10%) had a vegetarian or vegan diet (Table 1.) Use of a special omnivore diet (Fodmap, gluten-free, low-carb, stone age) or a vegetarian/vegan diet was more common among women (19%), than men (5%), p = 0.007. While the majority of the students had always had an omnivore diet (n = 299, 76%), almost one fourth of the students (n = 95, 24%) reported either former or current use of a vegetarian or vegan diet, also with a significant female dominance (28% of the women and 10% of the men, p < 0.001). Current vegetarians had lower BMI compared to omnivores (mean 22 (SD 2) versus mean 23 (SD 3), p = 0.04), whereas there was no difference in age between the two groups (p = 0.34).

### Use of supplements

More than half of the students (52%) reported regular intake of a nutritional supplement three or more days per week (Table 1). The most popular supplement was cod liver oil or omega 3 fatty acids, reportedly being used by 39% of the students, with no difference according to gender (Table 1) or between vegetarian/vegans (43%) versus omnivores (38%), p = 0.60.

A regular intake of multivitamins/ minerals was more common among women than men (Table 1) and among vegetarians/ vegans (70%) compared to omnivores (23%), p < 0.001. Use of iron supplement was also more common among women than men (Table 1), and among vegetarian/vegans (23%) versus omnivores (12%), p = 0.05.

### Dinner preferences

The majority of omnivore students reported eating dinner more than once a week and their food preferences are shown in Table 2. The most frequent choices for dinner were white meat, fatty fish and processed meat. The students reported eating fatty fish almost three times more often than lean fish, and 53% of the students never ate lean fish for dinner (Table 2). Men reported eating red meat for dinner more frequent than women (mean 1.6 (SD 1.3) days/week for men versus mean 0.9 (SD 0.9) days/week for women, p < 0.001) and the same was seen for processed meat (mean 1.7 (SD 0.9) days/week for men versus mean 1.3 (SD 0.8) days/week for women, p = 0.001). A vegetarian dinner was more common among omnivore women than men (mean 1.5 (SD 1.5) days/week for women versus mean 0.7 (SD 1.2) days/week for men, p < 0.001). No gender differences were seen for other dinner choices (Table 2).

**Table 2:** Food choices for dinner among medical students with an omnivore diet (n = 354)

How often do you have the following foods for dinner?	4–7 days/week*	1–3 days/week*	Never*
Red meat	9 (3%)	230 (65%)	115 (33%)
White meat	12 (3%)	311 (88%)	31 (9%)
Processed meat	4 (1%)	297 (84%)	53 (15%)
Fatty fish	4 (1%)	308 (87%)	42 (12%)
Lean fish	1 (0%)	164 (46%)	189 (53%)
Vegetarian dinner	35 (10%)	189 (53%)	130 (37%)
I do not eat dinner	2 (1%)	59 (17%)	293 (83%)

*Data are expressed as number of respondents (%).

### Food avoidance

Among the omnivores (n = 354), 70 students (20%) reported that they rarely or never ate meat, and this was more common among women (23%) than men (8%), p = 0.002. 14% of the omnivore students reported that they rarely or never ate fish, 8% rarely or never ate eggs and 3% rarely or never ate dairy products, with no gender differences observed for these foods (p > 0.41).

The most frequent causes given for rarely or never eating animal food among the omnivores were that the food was expensive (79%), unhealthy (70%) or they did not like it (61%). More infrequent causes were environmental concerns (38%), ethical concerns (27%), allergy (7%) and religious reasons (2%).

### Food attitudes

Student’s agreement to different statements about food are given in Table 3. The majority considered white meat to be healthier than red meat and fish to be healthier than meat. More women (67%) than men (49%) reported that they try to eat less meat, p = 0.01. No other significant differences in food attitudes according to gender were found. There was also no difference in the use of cod liver oil or omega 3 supplements between students who agreed to the statement “Fatty fish is healthier than lean fish” (Table 3) and students who did not (53% versus 44%, p = 0.61).

**Table 3:** Food attitudes among medical students (n = 394)

Food statements	Number (%) of respondents who agree
A vegetarian diet is healthier than an omnivore diet	82 (21%)
White meat (e.g. chicken) is healthier than red meat (e.g. beef)	326 (83%)
Fish is healthier than meat	252 (89%)
Fatty fish is healthier than lean fish	196 (50%)
I try to eat less meat	249 (63%)
I try to eat more fish	299 (76%)
There is a lot of media focus on eating less meat	315 (80%)
There is a lot of media focus on eating more fish	249 (63%)

## Discussion

The majority of the Norwegian medical students were omnivores, but exposed a negative attitude towards meat and considered fish to be healthier than meat. While fatty fish was a popular choice for dinner, more than half of the students reported that they never ate lean fish. Significantly more women than men reported use of a special diet and excluded meat from their diet, even if they were omnivores. The most frequently used supplement was cod liver oil or omega 3 fatty acids.

### Food habits and attitudes towards meat

While meat historically has played a central role as a symbol of higher social classes and wealth [14], there is a growing interest towards more sustainable eating behaviour from meat-centric to plant-based diets [15].

The term “cognitive vegetarians” has been used to describe those who hold similar beliefs about meat and vegetarian diets as do vegetarians and have lower red meat consumption compared to the general population, but do not consider themselves to be vegetarian [16]. Despite being omnivores, a negative attitude towards meat in general, but particularly red meat, was reported by the majority of the medical students, and women tended to be more negative than men. This is in accordance with published studies [17, 18]. According to a consumer report from 2017, 44% of consumers in Germany follow a low-meat diet, a significant increase from 2014 (26%), and 6% of US consumers claimed to be vegan, up from 1% to 2014 [19].

Female students reported more frequent use of a vegetarian, vegan or any type of special diet compared to their male colleagues, a finding which is in accordance with reports from other countries [18, 20, 21]. Omnivore women were more likely to exclude or limit the amount of meat in their diet than men. A similar avoidance of animal foods was reported in female Australian university students, and this was associated with a lower intake of omega-3 fatty acids, vitamin B12, selenium and zinc [22].

Male medical students reported eating red meat and processed meat for dinner more often than female students. The gender difference in meat consumption is well-known and is reported to start after the age of four, reaching a maximum between 51 and 65 years [23]. Among Norwegian men, meat has been reported to be associated with protein, masculinity and comfort [24], and these associations may contribute to the fact that men are more hesitant than women to reduce their intake of meat.

Most students considered white meat to be healthier than red meat. A British study has also found chicken and turkey to be the least often avoided flesh foods among women and men [25], and the growth in meat consumption is largely driven by white meats [26]. A large study published in 2012, concluded that red meat consumption was associated with an increased risk of total cardiovascular and cancer mortality [27]. The study has been much cited and was also a hit in the media. Overall, excess meat consumption, particularly red and processed meats, has in the latest years been associated with both nutritional [28] and environmental [29, 30] health harms. The data concerning red meat and negative health effects are however conflicting. Some researchers have not found an association between moderate red meat consumption and increased mortality from cardiovascular disease [31], and it has been shown that diets high in lean red meat can lower plasma cholesterol, contribute significantly to tissue omega-3 fatty acid and provide a good source of iron, zinc and vitamin B12 [32]. So whether red meat is unhealthy or not is still an unresolved issue, nevertheless, the recommendation from the health authorities has been to reduce the consumption of red meat [33], and our data show that the Norwegian medical students follow this advice.

### Food habits and attitudes towards fish

The majority (89%) of the students considered fish to be healthier than meat (Table 3), which is in accordance with current dietary Norwegian recommendations[12]. A similar finding was seen in a study from Belgium, where more than 90% of the respondents believed that fish consumption is healthy [18]. While fatty fish was a popular choice for dinner, more than half of the students reported that they never ate lean fish. This is in accordance with a recently published study, showing that Norwegian women aged 18 to 40 years most frequently ate farmed salmon (72%), followed by lean fish (17%) and other types of fatty fish (11%) [34]. The low intake of lean fish differs from data from other European countries. In a study from 2003 including data from 10 European countries, lean fish represented 49% and 45% of the total fish intake in women and men overall, and the greatest intake of very fatty fish was found in Denmark, Sweden, Norway and Germany [35].

### Use of supplements

More than half of the Norwegian medical students reported regular intake of a nutritional supplement, and cod liver oil or omega 3 supplements were most popular. Use of nutritional supplements is reported to be more common among medical students than those studying other disciplines [36, 37]. Regular use of multivitamins/ minerals supplements was more common among women than men, and among vegetarian/ vegans (70%) compared to omnivores (23%). Our data resemble both American and European reports. The American National Health and Nutrition Examination Survey (NHANES) study during 1999–2000; women, older age groups, and people with a higher education level, lower body mass index, higher physical activity level, and more frequent consumption of wine had a greater likelihood of reporting use of multivitamin-multimineral supplements [38]. The European Prospective Investigation into Cancer and Nutrition (EPIC)-Heidelberg study showed that individuals with a consistent intake of a vitamin and/or mineral supplement had the highest intake of dairy products, fish, fruits, vegetables and wine, but the lowest intake of total meat [39].

### Potential conflicts of interest in the concept of healthy food

Nutrition is not included as a separate course in the medical curriculium at the University of Bergen, but is integrated in the preclinical and clinical courses throughout the six years study. Students from all 6 years were evenly represented in our study population, and published results have shown that their nutritional knowledge about micronutrients did not differ significantly according to years of study [13], which may reflect a lack of nutritional education throughout the study. Also factors like marketing, environmental health and medicine-food industry interactions are given little attention in the Norwegian medical curriculum, and the students dietary attitudes may be affected by these factors.

For example, fatty fish and omega 3 supplements have been promoted like nutraceuticals since the 1970, when Danish researchers after having studied Inuit metabolism, proposed that omega-3 fatty acids found in fish were protective against cardiovascular disease [40]. However, a recent Science study showed that the Inuits were protected against CVD by their genes [41] and several reviews, including Cochrane reports, find no support for current recommendations to use omega-3 fatty acids for the prevention of heart disease, stroke or overall mortality [42, 43]. Despite this, omega 3 supplements continue to be promoted and are used by the consumers, which is reflected in our data.

The continued preference for fatty fish and omega 3 supplements among the Norwegian medical students may be related to the fact that Norway is the world’s largest producer of farmed salmon. In Norway, this is reflected in media marketing and clinical trials, of which several are funded by the seafood industry. Many, but not all of these, report positive effects of fatty fish intake [44–47]. On the other hand, a large independent Norwegian population based study, published in 2017, just a year before this survey, showed that lean fish consumption was significantly associated with better metabolic parameters and decreased waist circumference and blood pressure, while fatty fish consumption was significantly associated with increased waist circumference for both genders [48]. Also the independent Norwegian Mother and Child Cohort study, including more than 100 000 pregnancies, found that maternal lean fish intake during pregnancy was positively associated with all newborn birth size measures, while fatty fish was not related to any growth parameters and intake of supplementary n-3 was in fact negatively associated with newborn head circumference [49]. Particularly the latter finding was rather dramatic, it did however not get any media attention in Norway.

Food is also the main contributor of environmental pollutants and dietary exposure even of low levels in food is a health concern. Due to serious health effects caused by dioxins and dioxin like (dl) PCBs and perfluoroalkyl substances (PFAS) [50, 51], the European Food Safety Authority (EFSA) have reduced the tolerable weekly intake (TWI) of these persistent organic pollutants [52] several times. The main contributors to dietary exposure of dioxins and dl PCBs and PFAS are fish and in particular fatty fish [53], which were one of the preferred dinner-choices to most of the students. In Norway, 100% of children aged 2 to 9 years have a dietary intake of dioxins and dl PCBs which is higher than the current TWI and adults have an intake of PFAS which is 1.7 times higher than the current TWI for PFAS. Despite this, the Norwegian Scientific Committee for Food and Environment recommended in 2022 all age groups to increase their intake of fish [54].

Also advisory panel members may be influenced by the food industry [55], and medical doctors need adequate nutritional knowledge to evaluate if national dietary recommendations are optimal. Environmental medicine needs to be integrated into the medical curriculum, and studies have shown that this increases medical students’ preparedness to discuss environmental health issues [56].

The food industry has been funding nutrition research and has donated millions of dollars to universities and other academic programs over the years [7]. Such funding can create conflicts of interest in research, teaching and clinical practice. Addressing conflicts of interest around nutritional advices is pivotal to ensure that these are free from commercial and other vested interests of unhealthy commodities [57]. Studies have demonstrated that it is possible to improve knowledge and scepticism among medical students towards influence from the pharmaceutical industry [58]. It is just as important to educate medical students about marketing and medicine-industry interactions related to the food industry, in order to enhance nutritional understanding and make physicians capable of giving adequate nutritional counselling to their patients [59].

### Study limitations

The survey had a low response rate (45%), which reduces the generalizability. A strength of the survey is that students from all six years were evenly represented. The questionnaire has not been validated and included multiple choice questions which may induce response bias, i.e. the tendency to respond inaccurately or falsely. Based on these limitations, further research on physicians’ attitudes towards different diets and their impact on patient care outcomes is obviously needed.

## Conclusion

Our results suggest that dietary habits and food attitudes among Norwegian medical students not only reflect current health recommendations, but also popular beliefs, media coverage and marketing. Adequate nutritional education is important to ensure that future doctors can give non-biased scientific nutritional counselling to their patients. Curriculum planners should address environmental medicine, common attitudes and beliefs about nutrition in order to make medical doctors capable of recognizing and discussing the complex influence of social media, marketing and medicine-food industry interactions.

## Data Availability

Both authors have full access to the data reported in the manuscript, and this material will be made available on reasonable request.
